# Supplementary material for: Tuning of motor outputs produced by spinal stimulation during voluntary control of torque directions in monkeys
Source: eLife. 2022 Dec 13;11:e78346. doi: 10.7554/eLife.78346 (PMC9747157; doi:10.7554/eLife.78346)
Supplement: Supplementary file 1. [file elife-78346-supp1.docx]

**Supplementary File 1**

**Tuning of motor outputs produced by spinal stimulation during voluntary control of torque directions in monkeys**

Miki Kaneshige, Kei Obara, Michiaki Suzuki, Toshiki Tazoe, Yukio Nishimura

**Table 1. Summary of collected muscular conditions**

| Stim. site | Monkey H | | | |  | Monkey W | | | |  | Total |
| --- | --- | --- | --- | --- | --- | --- | --- | --- | --- | --- | --- |
|  | <150 μA | 150-750 μA | 750-1350 μA | ≥1350 μA |  | <150 μA | 150-750 μA | 750-1350 μA | ≥1350 μA |  |  |
| 1 | 16 | 64 | 48 | 0 |  | 64 | 16 | 0 | 0 |  | 208 |
| 2 | 0 | 16 | 0 | 0 |  | 32 | 16 | 0 | 0 |  | 64 |
| 3 | 0 | 48 | 48 | 0 |  | 16 | 48 | 16 | 16 |  | 192 |
| 4 | 32 | 32 | 0 | 0 |  | 32 | 48 | 32 | 0 |  | 192 |
| 5 | 16 | 48 | 16 | 0 |  | 48 | 0 | 0 | 0 |  | 128 |
| 6 | 16 | 16 | 48 | 0 |  | 0 | 48 | 0 | 0 |  | 128 |
| 7 | 16 | 16 | 48 | 16 |  |  | | | |  | 96 |
| Total | 96 | 240 | 208 | 16 |  | 156 | 156 | 48 | 16 |  | 1008 |

**Table 2. Summary of collected experiments and trials**

| Stim. site | Monkey H | | | |  | Monkey W | | | |  | Total experiments  (min-max trials) |
| --- | --- | --- | --- | --- | --- | --- | --- | --- | --- | --- | --- |
|  | <150  μA | 150-  750  μA | 750-1350  μA | ≥1350 μA |  | <150  μA | 150-  750  μA | 750-  1350  μA | ≥1350 μA |  |  |
| 1 | 1  (872) | 4  (250-303) | 3  (255-279) | 0  (0) |  | 4  (102-251) | 1  (112) | 0  (0) | 0  (0) |  | 13  (102-872) |
| 2 | 0  (0) | 1  (321) | 0  (0) | 0  (0) |  | 2  (209-226) | 1  (113) | 0  (0) | 0  (0) |  | 4  (113-321) |
| 3 | 0  (0) | 3  (105-248) | 3  (129-301) | 0  (0) |  | 1  (299) | 3  (103-400) | 1  (200) | 1  (101) |  | 12  (101-400) |
| 4 | 2  (125-867) | 2  (294-307) | 0  (0) | 0  (0) |  | 2  (245-246) | 4  (194-228) | 2  (79-  166) | 0  (0) |  | 12  (79-867) |
| 5 | 1  (810) | 3  (180-300) | 1  (304) | 0  (0) |  | 3  (63-212) | 0  (0) | 0  (0) | 0  (0) |  | 8  (63-810) |
| 6 | 1  (592) | 1  (266) | 3  (248-279) | 0  (0) |  | 0  (0) | 3  (204-248) | 0  (0) | 0  (0) |  | 8  (204-592) |
| 7 | 1  (1004) | 1  (251) | 3  (261-395) | 1  (74) |  |  | | | |  | 6  (74-1004) |
| Total experiments  (min-max trials) | 6  (125-1004) | 15  (105-321) | 13  (129-395) | 1  (74) |  | 12  (63-299) | 12  (103-400) | 3  (79-200) | 1  (101) |  | 63  (63-1004) |
